# Increase in *Kelch 13* Polymorphisms in *Plasmodium falciparum*, Southern Rwanda

**DOI:** 10.3201/eid2701.203527

**Published:** 2021-01

**Authors:** Clara Bergmann, Welmoed van Loon, Felix Habarugira, Costanza Tacoli, Julia C. Jäger, Darius Savelsberg, Fabian Nshimiyimana, Elias Rwamugema, Djibril Mbarushimana, Jules Ndoli, Augustin Sendegeya, Claude Bayingana, Frank P. Mockenhaupt

**Affiliations:** Charité–Universitätsmedizin Berlin, Berlin, Germany (C. Bergmann, W. van Loon, C. Tacoli, J.C. Jäger, D. Savelsberg, F.P. Mockenhaupt);; University Teaching Hospital of Butare, Butare, Rwanda (F. Habarugira, E. Rwamugema, D. Mbarushimana, J. Ndoli, A. Sendegeya);; Kabutare District Hospital, Butare (F. Nshimiyimana);; University of Rwanda, Kigali, Rwanda (C. Bayingana)

**Keywords:** antimicrobial resistance, artemisinin, Kelch, malaria, mutation, parasites, Plasmodium falciparum, polymorphism, vector-borne infections

## Abstract

Artemisinin resistance in *Plasmodium falciparum* is associated with nonsynonymous mutations in the *Kelch 13* (*K13*) propeller domain. We found that 12.1% (8/66) of clinical *P. falciparum* isolates from Huye district, Rwanda, exhibited *K13* mutations, including R561H, a validated resistance marker. *K13* mutations appear to be increasing in this region.

Emerging artemisinin resistance to *Plasmodium falciparum* endangers malaria control worldwide. Currently, the resistance epicenter is the greater Mekong subregion in Southeast Asia (*1*). In sub-Saharan Africa, where illnesses and deaths from *P. falciparum* malaria are highest, such resistance may result in disastrous consequences (*2*). Early detection and close monitoring are therefore crucial. 

Artemisinin resistance in *P. falciparum* is associated with *pfkelch13* polymorphisms encoding the parasite’s *Kelch 13* (*K13*) propeller domain, which consequently serve as a molecular marker in surveillance (*3*). More than 200 nonsynonymous *K13* single-nucleotide polymorphisms have been reported, including 11 candidate resistance mutations (i.e., associated with delayed parasite clearance) and 9 validated mutations (i.e., also reduced in vitro sensitivity) (*4*). Compared with those from Asia, isolates from sub-Saharan Africa show pronounced heterogeneity of nonsynonymous *K13* polymorphisms, most of them rare, possibly reflecting lower drug pressure (*5*). 

Rwanda achieved substantial reductions in malaria during 2006–2011, partly due to home-based management using artemether/lumefantrine (*6*). In 2010, at our study site in Huye district, southern Rwanda, we observed a pattern in the *P. falciparum* multidrug resistance: 1 gene suggestive of intense artemether/lumefantrine drug pressure, whereas *K13* mutations were absent. However, among *P. falciparum* isolates, 2.5% in 2014 and 4.5% in 2015 harbored *K13* variants, including 2 candidate mutations (*7*,*8*). A recent report showed the presence of a validated *pfkelch13* mutation, R561H, at 2 sites in Rwanda (*9*). We conducted a cross-sectional molecular surveillance study to update records of the prevalence of *K13* variants in Huye among isolates collected in 2019.

## The Study

During September–December 2019, we recruited study patients with uncomplicated malaria seeking treatment at the Sovu Health Centre and Kabutare District Hospital, Huye district, Rwanda. Huye district (population ≈390,000) is located on the central plateau of Rwanda (average altitude 1,700 m, yearly rainfall 1,200 mm, mean temperature 19°C). Malaria transmission peaks in October–November and March–May. In 2010, a total of 11.7% of children had microscopically confirmed *Plasmodium* infection (*8*). 

We obtained written informed consent from all participants or from the caregivers for children; we also obtained written assent from participants 7–18 years of age. The study was approved by the Rwanda National Ethics Committee. Eligibility criteria for participants included age >1 year; a positive result on a rapid diagnostic test, SD Bioline Malaria Ag Pf/Pan (Abbott Global Point of Care, https://www.globalpointofcare.abbott); and a fever (axillary temperature ≥37.5°C) at the time they sought treatment or within 48 hours beforehand (self-reported). We collected whole blood in S-Monovette EDTA (ethylenediaminetetraacetic acid; Sarstedt, https://www.sarstedt.com) tubes and confirmed malaria diagnosis by microscopy of Giemsa-stained thick blood smears; patients were also seen by a physician. We provided a 3-day regimen of artemether/lumefantrine for treatment, the first dose given under observation. All patients were asked to return after 3 days to evaluate residual parasitemia on Giemsa-stained thick blood smears.

Definite parasite density was counted per 200 leukocytes on Giemsa-stained thick blood smears by 2 independent microscopists, assuming a mean leukocyte count of 8,000/µL. We extracted DNA using a QIAamp DNA Blood Mini kit (QIAGEN, https://www.qiagen.com). *Plasmodium* species were typed by real-time PCRs with commercially available primers and probes for *P. falciparum*, *P. vivax*, *P. ovale*, *P. malariae*, and *P. knowlesi* (TIB MolBiol, https://www.tib-molbiol.com) on a Roche LightCycler 480 device (https://lifescience.roche.com). *K13* was amplified (codons ≥441≤688) by using nested PCR (*3*) and sequenced by a commercial provider (Eurofins Genomics, https://www.eurofinsgenomics.com). Sequences were aligned to reference *K13* 3D7–1343700 (PlasmoDB, https://plasmodb.org) by using Geneious Prime version 2020.1 (https://www.geneious.com). We used R version 3.6.3 (https://cran.r-project.org) for statistical analysis and a binomial logistic regression model to estimate the time-trend of nonsynonymous mutations (p<0.05). 

Of 90 patients included in the study, 74 tested positive by microscopy and PCR and 2 by PCR only. Among these patients, 51.3% (39/76) were female and 4 were pregnant; the median age was 18 years (range 2–69 years). *P. falciparum* was found in 88.2% (67/76), *P. vivax* in 7.9% (6/76), *P. ovale* in 7.9% (6/76) and *P. malariae* in 1.3% (1/76). The geometric mean parasite density, based on microscopy results, was 8,926 parasites/µL (95% CI 5,911–13,478 parasites/µL); mean temperature was 37.4°C (SD ±1.3°C). After 3 days of treatment, 61 malaria patients had a negative blood smear, 1 patient (1.6%, 1/62) had asymptomatic parasitemia (31,520 parasites/µL), and 14 patients did not return for the day 3 checkup. 

None of the patients infected with *K13* variant parasites tested positive after 3 days of treatment. One pregnant patient sought treatment again. Initially, she had *K13* wild-type parasites and was given artemether/lumefantrine; her day 3 microscopy result was negative. Three weeks later, we detected *K13* R561H parasites, possibly due to reinfection, and administered quinine.

Samples from 98% (66/67) of *P. falciparum* isolates were successfully sequenced for the *K13* propeller domain. We found 5 different nonsynonymous polymorphisms in 8 isolates (Table); 3 harbored R561H, a validated resistance mutation, and the 2 candidate polymorphisms C469F and A675V (*4*). This finding suggests that the number of isolates with nonsynonymous *K13* mutations had increased significantly over the previous decade (OR 1.4, 95% CI 1.1–1.8; p = 0.003). 

**Table:** Nonsynonymous single nucleotide polymorphisms in the *Kelch 13* propeller domain of clinical *Plasmodium falciparum* isolates collected in the Huye District, Rwanda, 2010–2019*

Year	No. sequenced isolates	No. (%) isolates with nonsynonymous mutations	Amino acid changes and nucleotide changes
2010	75	0	Not applicable
2014	81	2 (2.5)	V555A, A626S
2015	66	3 (4.5)	P574L,† D648H, A675V†
2019	66	8 (12.1)	C469F,† G533A, V555A, R561H‡ (3×), A578S, A675V†

*Data during 2010–2015 derived from Tacoli et al. (*7*).  †Candidate mutations for artemisinin resistance.  ‡Validated mutation for artemisinin resistance (*4*).

## Conclusions 

Of *P. falciparum* isolates from symptomatic patients in southern Rwanda, 12% exhibited nonsynonymous *K13* mutations, a significant increase (OR 1.4, 95% CI 1.1–1.8; p = 0.003) over the previous decade compared with their absence in 2010 and 4.5% prevalence in 2015 (*7*). Of note, the validated marker R561H alone occurred in 4.5% of the isolates collected in 2019. Recent studies report 1%–3.5% of nonsynonymous *K13* polymorphisms in parasite isolates from East Africa (*10*), whereas during 2013–2015 in Rwanda, this figure was 6.9% (*9*). 

The R561H artemisinin resistance mutation is regularly observed across Asia (*10*). A recent study that reported R561H in 7.4% of isolates collected during 2013–2015 in central Rwanda and 0.7% of isolates in south-central Rwanda (*9*) suggested that this mutation emerged indigenously and independently from Asia 561H strains. We do not have data in our study to support this. None of the *K13* variant parasites showed delayed clearance in our study, which may be due to the partner drug lumefantrine still being effective, similar to observations in Southeast Asia (*11*). In addition, the absence of delayed parasite clearance despite *K13* mutations may reflect partial immunity contributing to parasite elimination (*12*). 

We found other nonsynonymous polymorphisms only once among the isolates tested. C469F and A675V are considered artemisinin resistance candidate mutations (*4*) and have previously been seen in East Africa (*7*,*13*,*14*). G533A and V555A have also been previously reported in Africa but have not yet been evaluated for resistance (*7*,*13*). A578S is a common *K13* polymorphism across Africa but is not linked to artemisinin resistance (*1*). 

Our study has clear limitations. Data from only 2 healthcare facilities, with limited catchment areas, were included. Adherence to treatment was assessed by patient self-report, and drug susceptibility testing was not performed. Future research should include ring-stage susceptibility assays to contribute to understanding the role of *K13* mutations in Africa. Separate testing for each drug in a combination for efficacy and continued surveillance for antimicrobial resistance are needed.

Our results show that *K13* mutations are present in Rwanda and that their prevalence in *P. falciparum* malaria patients in the Huye District increased from 0% in 2010 to >12% in 2019. The validated artemisinin resistance mutation R561H occurs in 4.5% of *P. falciparum* isolates being transmitted in this area. The emergence of artemisinin resistance–related mutations in Rwanda is alarming because it might indicate developing resistance against commonly used antimalarials in this region. Countermeasures need to be considered early, potentially including 3-drug antimalarial combinations (*2*). 
